# Smart Speakers: The Next Frontier in mHealth

**DOI:** 10.2196/28686

**Published:** 2022-02-21

**Authors:** Jacob Sunshine

**Affiliations:** 1 Department of Anesthesiology & Pain Medicine University of Washington Seattle, WA United States; 2 Paul G Allen School of Computer Science and Engineering University of Washington Seattle, WA United States

**Keywords:** digital health, mobile health, machine learning, smart speaker, smartphone

## Abstract

The rapid dissemination and adoption of smart speakers has enabled substantial opportunities to improve human health. Just as the introduction of the mobile phone led to considerable health innovation, smart speaker computing systems carry several unique advantages that have the potential to catalyze new fields of health research, particularly in out-of-hospital environments. The recent rise and ubiquity of these smart computing systems holds significant potential for enhancing chronic disease management, enabling passive identification of unwitnessed medical emergencies, detecting subtle changes in human behavior and cognition, limiting isolation, and potentially allowing widespread, passive, remote monitoring of respiratory diseases that impact public health. There are 3 broad mechanisms for how a smart speaker can interact with a person to improve health. These include (1) as an intelligent conversational agent, (2) as a passive identifier of medically relevant diagnostic sounds, and (3) by active sensing using the device's internal hardware to measure physiologic parameters, such as with active sonar, radar, or computer vision. Each of these different modalities has specific clinical use cases, all of which need to be balanced against potential privacy concerns, equity concerns related to system access, and regulatory frameworks which have not yet been developed for this unique type of passive data collection.

## Background

The rapid dissemination and adoption of smart speakers has enabled substantial opportunities to improve human health. Just as the introduction of the mobile phone led to considerable health innovation, and ultrasound enabled new opportunities for point-of-care diagnosis and procedural optimization, smart speaker computing systems carry several unique advantages that can catalyze new fields of research, particularly in out-of-hospital environments. The recent rise and ubiquity of these smart computing systems, which are often cheaper than smartphones and substantially less expensive than medical grade equipment, holds significant potential for enhancing chronic disease management, enabling passive identification of unwitnessed medical emergencies, detecting subtle changes in human behavior and cognition, limiting isolation, and potentially allowing widespread, passive, remote monitoring of respiratory-based infectious diseases which impact public health, all while still providing general utility for users. Advances in machine-based classification of disease states, capable of being run on-device and securely in the cloud, can enable rapid diagnostic and predictive functions at a low cost while preserving privacy. This confluence of factors has created a significant opportunity involving these devices, which currently reside in 1 of 4 US households, when applied thoughtfully to carefully chosen health conditions [1].

## What Are Smart Speakers and How Are They Different From Smartphones?

At its most basic form, a smart speaker is a system comprising a speaker, a microphone array, an embedded computer, a software- and machine learning–based intelligent assistant, and wireless connectivity that enables data integration with the cloud, nearby smart devices, and other information technology (IT) infrastructures outside of the home. The increasing computational horsepower of embedded platforms coupled with advances in machine learning have enabled on-device capabilities that remove the need to transmit audio to the cloud. As such, the system has the capability to continuously monitor the home environment and instruct a patient on or converse with them about a medically relevant topic, identify health-related audible biomarkers, sense the environment for contextually relevant health-related motion, and much more. And because these computing systems have wireless capability, they can transmit data to the cloud for secure storage and analysis, if desired. Such connectivity also, in theory, enables integration with medical IT infrastructures, so a trained provider can interpret, triage, and act upon relevant information from a smart speaker, or in an emergent context, connect with an emergency response system (eg, 911) to summon help. Key differentiators of these devices compared to mobile phones include that they are plugged in, thus avoiding power constraints that are associated with charging a device; they are predominantly stationary, enabling long-term, passive, and continuous monitoring; and their range of measurements is greater than a phone, which generally must be interacted with when it is directly in a user’s hands. The inherent constraints of their placement, moreover, provide a substantive benefit by reducing the number of “edge cases” that invariably arise when building intelligent sensing systems. Yet, perhaps smart speakers’ biggest advantage over mobile phones and other wearable devices is their ability to foster compliance [2,3] by not requiring patients to wear or do anything after initial setup (ie, they can be truly “set and forget”). 

**Figure 1:**
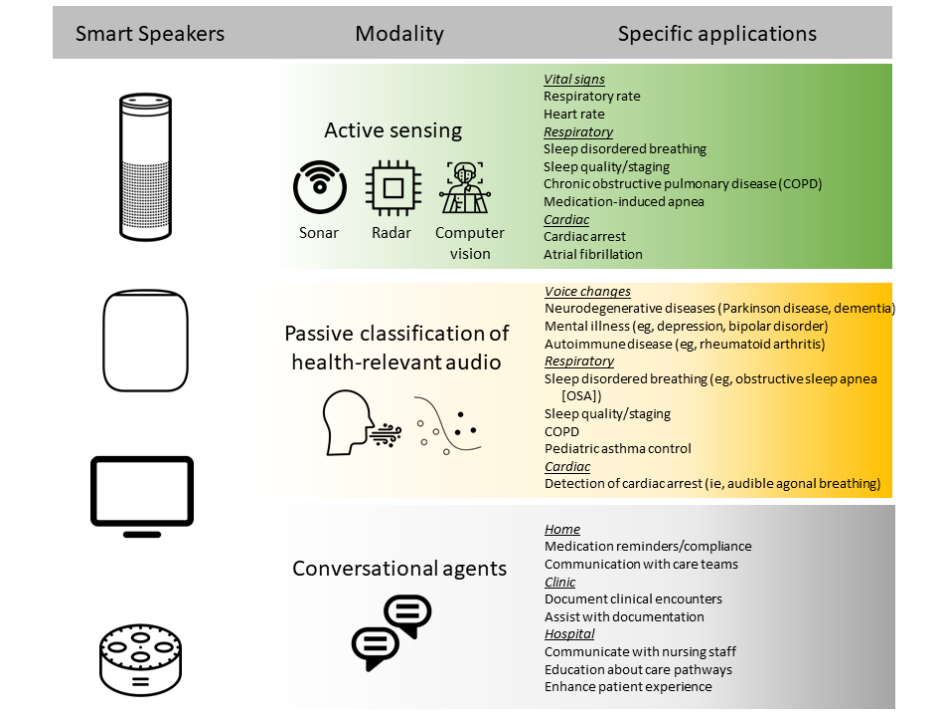
Overview of how smart speakers can enhance health and well-being.

Against this background, there are 3 broad mechanisms for how a smart speaker can interact with a patient to improve health. These include (1) as an intelligent conversational agent, (2) as a passive identifier of medically relevant diagnostic sounds, and (3) by active sensing using the device’s internal hardware to measure physiologic parameters, such as with active sonar, radar, or computer vision (Figure 1).

## Smart Speakers as Health Conversation Agents

The first deployed and most straightforward use for smart speakers is as intelligent conversational agents and facilitators. These applications generally rely on voice user interfaces (VUIs), which enable the user to interact with the system using their voice and allow them to receive medically relevant auditory feedback [4]. In the home environment, conversational use cases include the system providing reminders to take medications, retrieving recent lab results (eg, blood sugar), managing medical appointments, and tracking wellness goals [5]. These systems are also capable of reducing isolation, particularly in older adults, by providing a low-barrier way to facilitate communication (eg, with family members, caretakers, social workers), and detecting signals in the environment where a check-in may be warranted (eg, a change or reduction in activities of daily living). Outside of the home, these devices also have a role in the clinic and the inpatient environment. Within the clinic, these devices may soon be used to help liberate physicians from their computers, as provider-patient conversations are passively captured, parsed, and analyzed to efficiently document medical encounters [6]. Devices have also been deployed in hospitals, particularly in patient rooms, primarily as a way to improve the patient experience [7], and in the era of COVID-19, to provide a crucial means of communication with the care team and family members unable to visit the patient [8].

## Classification of Medically Relevant Diagnostic Sounds

The next level of interaction with these devices is as a classifier of medically relevant, contextually appropriate biosignals that represent signs and symptoms of disease. There have been major advances in sound classification research in the computing community [9-11] that have implications for medically informative audio [12]. Researchers are examining publicly available data sets from the computing community, such as AudioSet [13], to relabel and train new models for medically relevant sounds [14]. In this use case, which would predominate in home environments, the computing system classifies certain audible biomarkers for the purposes of diagnosis or to better inform disease management. Similar to invoking certain trigger words (eg, “Hey Siri,” “Alexa,” “Hey Bixby,” “OK, Google”), these systems are capable of passively identifying specific audio signatures that are contextually relevant and of medical utility. Building on classification guidance from the National Institutes of Health (NIH) and the US Food and Drug Administration (FDA), Coravos et al [15] have proposed a useful framework of digital biomarkers, which classifies signals as they relate to susceptibility or risk, diagnosis, monitoring, prognostication, and prediction. These audio biomarkers can be used to detect and classify coughs [16,17], discern voice changes arising from neurodegenerative diseases such as Parkinson disease [18] or dementia [19], characterize voice changes related to depression [20,21] or other mental illnesses [22], classify breathing patterns associated with obstructive sleep apnea (OSA) [23], identify deteriorating asthma [24], and even identify unwitnessed cardiac arrest by detecting the presence of agonal breathing [11].

## Active Sensing Using Smart Devices

The final way that these computing systems can be used is perhaps the most innovative and involves turning these devices into contactless active sensing systems using computer vision, sonar, or radar for the purposes of physiologic monitoring. If the smart speaker has a camera, this enables important diagnostic capabilities aided by computer vision, which enables a machine to make inferences based on dynamic images and subtle changes in pixelation. Notable potential use cases for computer vision include the detection of falls [25], respiratory and heart rate monitoring [26,27], identifying significant changes in activity in older populations [28], self-monitoring of physical therapy, monitoring of acute and chronic wounds [29], and postoperative- and posthospitalization-based rehabilitation within the home. In addition, because these devices have speakers and microphones, they are capable of active sonar and echolocation utilizing high (>18 kHz), inaudible frequencies to detect medically relevant motion. Some smart speakers are already enabling these features for activity sensing and gesture detection. A benefit of this method is that, because it utilizes inaudible frequencies, it can collect relevant data while filtering out all audible speech and thus preserves privacy. Similarly, in radar-based systems, electromagnetic waves are transmitted into the environment and phase changes in the reflected signals can be used to classify medically relevant motion. The potential use cases of these sonar- and radar-based active sensing modalities include monitoring of chest motion or breathing [30] and its perturbations (pertinent for asthma [31], chronic obstructive pulmonary disease [COPD] [32], OSA [33], and opioid overdose [34]), sleep disturbance (eg, insomnia), identification of incipient respiratory infection, measurement of cardiac activity (eg, heart rate and atrial fibrillation) [35], monitoring of activity levels based on movement, epilepsy monitoring, and more.

## Privacy

As with any ubiquitous computing system, a critical consideration relates to privacy, which can mean different things to different people. For a health monitoring context, this refers to monitoring that, similar to the default functionality of these devices, enables continuous “listening,” but only processes and stores (if the user desires) relevant health data. In practice, using asthma or COPD as an example, the system would not store or analyze conversations, though it would recognize, document, and analyze increases in nocturnal cough or relevant changes in respiration, such as dyspnea or audible wheezing. It is important that any health-related data approved to be stored are stored securely within an environment designed to be compliant with the Health Insurance Portability and Accountability Act (HIPAA) and the General Data Protection Regulation (GPDR), and that the data belong to and are made easily accessible to individuals. Just as there are potential privacy concerns associated with smartphones and personal computers, there is a point where their utility outweighs their real and perceived privacy concerns. The adoption arc for smart speakers is undoubtedly affected by these concerns, presenting a challenge but also an opportunity to develop innovative privacy-preserving functionality that would make the collection of health data more comfortable and trustworthy. These efforts would be greatly enhanced by manufacturers taking straightforward, transparent actions that foster trust and maximize control of information for the monitored user.

## Barriers to Implementation and Future Directions

Although there is tremendous potential for this new computing platform to potentially improve human health, there remain several barriers. The first major barrier is the lack of an open ecosystem, compared to the development environment and regulatory frameworks for applications that can run on smartphones, tablets, or PCs. Crucially, there is no app store or developer environment that provides the level of access to firmware that would enable flexible development of innovative, high-quality, medically relevant applications which take advantage of a device’s internal hardware. For example, unlike on Android or iOS, a developer cannot leverage the smart speaker’s camera, individual speaker(s), or microphones for the purposes of app development. Although the major smart speaker manufactures allow for the development of “skills” or plug-ins within a highly constrained design framework, including at least one enabling secure transmission of health information [36], they do not offer the openness and flexibility that exists for the development of health-related applications intended for smartphones. Such an ecosystem would represent a substantial opportunity for health-related software development and would leverage these devices’ full computational capabilities.

Control of data flow for regulatory and HIPAA standards is also critical in health care use cases. Regulatory organizations, health system stakeholders, and computing communities need to come together to develop an agreement on the responsible use of data for these emerging technologies. In particular, it is unclear what protections are needed for data generated in the home that *could* be used for health purposes compared to data that is generated in a clinic or hospital encounter, where protections are clearly enumerated for patient data. Current regulatory guidance does not take into account these new sources of data generated in the home, which will have to be addressed as these computing systems become more common for health purposes. Relatedly, thoughtful care must be taken when using voice or medically relevant audio as a passively measured biomarker. Such measurements are primarily relevant to the intended monitored user, who would have consented to these biosignals being collected, processed, and stored. Yet, such a design has implications when others are in close proximity to these systems because their biosignals could be captured without having provided explicit consent. Although there are several examples of people being monitored in everyday life without their explicit consent (eg, security-based audiovisual observation or being in the presence of others’ smart devices), passive health sensing must be undertaken with particular care given the nature of the data being collected.

Another critical consideration with passive systems deployed on ubiquitous devices is the need to minimize false positives. Generally, it is not wise to use these systems for asymptomatic screening of healthy populations given the dangers of excessive false positives. Using these systems to monitor specific patient populations at risk for certain physiologic perturbations that are clinically meaningful is more likely to be useful to the patient and care teams generally. Toward this end, following identification of a given biomarker or aberrant trend, effective uses of these systems will likely require a level of interactivity (via screen or voice) to collect further information, such as pertinent positives and negatives, before consequential actions or referrals are executed. Additionally, as these computing systems mature as tools for research, they will require a research platform that can enable vetted, high-quality studies at scale, similar to Apple’s ResearchKit, Sage Bionetwork’s Bridge Platform, and CareEvolution’s MyDataHelps. Such research is essential to demonstrate the health utility of these platforms, which will require actual clinical evidence to gain trust from patients, care teams and health systems. Finally, when used for health purposes, it is essential these devices do not exacerbate health disparities, for example, by being differentially accessible to certain populations. Concrete ways to reduce inequities include programs that make smart speakers, when indicated, accessible to those who desire them but may not be able to afford the cost. Similarly, if used for health purposes and prescribed by a care team, these systems should be readily covered by payers. Lastly, it is imperative that application VUIs and non-VUIs encompass as many languages as possible and, particularly for VUIs, that performance differences across language, age, sex, and gender are actively minimized and eventually eliminated.

## Conclusion

In summary, smart speakers represent a new, ubiquitous computing platform within our home environments, which hold considerable untapped potential to improve human health at low cost, and if done thoughtfully, in ways that foster high compliance and preserve privacy. The primary health benefits are likely to be observed with enhanced chronic disease management, early detection of unwitnessed emergencies and indolent neurodegenerative processes, and enhancements of the patient and provider experience in clinic and inpatient environments. Achieving this unrealized potential will require smart speaker manufacturers to open their platforms to developers as they have with smartphones, develop an ecosystem specifically for medically oriented applications and research, and enable and relentlessly prioritize privacy-preserving functionality. 

## Abbreviations

COPD: chronic obstructive pulmonary disease
FDA: US Food and Drug Administration
GPDR: General Data Protection Regulation
HIPAA: Health Insurance Portability and Accountability Act
IT: informational technology
NIH: National Institutes of Health
OSA: obstructive sleep apnea
VUI: voice user interface

## References

[1] The smart audio report. National Public Media.

[2] Hamine S, Gerth-Guyette E, Faulx D, Green BB, Ginsburg AS (2015). Impact of mHealth chronic disease management on treatment adherence and patient outcomes: a systematic review. J Med Internet Res.

[3] Galarnyk M, Quer G, McLaughlin K, Ariniello L, Steinhubl SR (2019). Usability of a wrist-worn smartwatch in a direct-to-participant randomized pragmatic clinical trial. Digit Biomark.

[4] Stigall B, Waycott J, Baker S, Caine K (2019). Older adults' perception and use of voice user interfaces: a preliminary review of the computing literature. Proceedings of the 31st Australian Conference on Human-Computer-Interaction.

[5] Jiang R (2019). Introducing new Alexa healthcare skills. Amazon Developer Services and Technologies: Amazon Alexa.

[6] Langston J (2019). Microsoft and Nuance join forces in quest to help doctors turn their focus back to patients. Official Microsoft Blog.

[7] Dietsche E (2019). Pilot project brings Alexa to Cedars-Sinai patients. MedCity News.

[8] Sezgin E, Huang Y, Ramtekkar U, Lin S (2020). Readiness for voice assistants to support healthcare delivery during a health crisis and pandemic. NPJ Digit Med.

[9] Siri Team (2017). Hey siri: an on-device DNN-powered voice trigger for Apple's personal assistant. Apple Machine Learning Research.

[10] Models for Audioset: a large scale dataset of audio events. GitHub.

[11] Muda L, Begam M, Elamvazuthi I (2010). Voice recognition algorithms using mel frequency cepstral coefficient (MFCC) and dynamic time warping (DTW) techniques. J Comput.

[12] Chan J, Rea T, Gollakota S, Sunshine JE (2019). Contactless cardiac arrest detection using smart devices. NPJ Digit Med.

[13] Gemmeke J, Ellis D, Freedman D, Jansen A, Lawrence W, Moore R, Plakal M, Ritter M (2017). Audio Set: an ontology and human-labeled dataset for audio events.

[14] Al Hossain F, Lover AA, Corey GA, Reich NG, Rahman T (2020). FluSense: a contactless syndromic surveillance platform for influenza-like illness in hospital waiting areas. Proceedings of the ACM on Interactive, Mobile, Wearable and Ubiquitous Technologies.

[15] Coravos A, Khozin S, Mandl KD (2019). Developing and adopting safe and effective digital biomarkers to improve patient outcomes. NPJ Digit Med.

[16] Larson E, Lee T, Liu S, Rosenfeld M, Patel S (2011). Accurate and privacy preserving cough sensing using a low-cost microphone. Proceedings of the 13th International Conference on Ubiquitous Computing.

[17] Sun X, Lu Z, Hu W, Cao G (2015). SymDetector: detecting sound-related respiratory symptoms using smartphones. Proceedings of the 2015 ACM International Joint Conference on Pervasive and Ubiquitous Computing.

[18] Bot BM, Suver C, Neto EC, Kellen M, Klein A, Bare C, Doerr M, Pratap A, Wilbanks J, Dorsey ER, Friend SH, Trister AD (2016). The mPower study, Parkinson disease mobile data collected using ResearchKit. Sci Data.

[19] Chen R, Jankovic F, Marinsek N, Foschini L, Kourtis L, Signorini A, Pugh M, Shen J, Yaari R, Maljkovic V, Sunga M (2019). Developing measures of cognitive impairment in the real world from consumer-grade multimodal sensor streams. Proceedings of the 25th ACM SIGKDD International Conference on Knowledge Discovery & Data Mining.

[20] Tasnim M, Stroulia E (2019). Detecting depression from voice. Advances in Artificial Intelligence.

[21] Huang Z, Epps J, Joachim D, Chen M (2018). Depression detection from short utterances via diverse smartphones in natural environmental conditions.

[22] Faurholt-Jepsen M, Busk J, Frost M, Vinberg M, Christensen EM, Winther O, Bardram JE, Kessing LV (2016). Voice analysis as an objective state marker in bipolar disorder. Transl Psychiatry.

[23] Dafna E, Tarasiuk A, Zigel Y (2018). Sleep staging using nocturnal sound analysis. Sci Rep.

[24] Larson E, Goel M, Boriello G, Heltshe S, Rosenfeld M, Patel S (2012). SpiroSmart: using a microphone to measure lung function on a mobile phone. Proceedings of the 2012 ACM Conference on Ubiquitous Computing.

[25] Anderson D, Keller J, Skubic M, Chen X, He Z (2006). Recognizing falls from silhouettes.

[26] Chatterjee A, Prathosh A, Praveena P (2016). Real-time respiration rate measurement from thoracoabdominal movement with a consumer grade camera.

[27] Yan BP, Lai WHS, Chan CKY, Au ACK, Freedman B, Poh YC, Poh M (2020). High-throughput, contact-free detection of atrial fibrillation from video with deep learning. JAMA Cardiol.

[28] Luo Z, Hsieh JT, Balachandar N, Yeung S, Pusiol G, Luxenberg J, Li G, Li LJ, Downing NL, Milstein A, Fei-Fei L (2018). Computer vision-based descriptive analytics of seniors' daily activities for long-term health monitoring. Proceedings of Machine Learning Research.

[29] Gunter R, Fernandes-Taylor S, Mahnke A, Awoyinka L, Schroeder C, Wiseman J, Sullivan S, Bennett K, Greenberg C, Kent KC (2016). Evaluating patient usability of an image-based mobile health platform for postoperative wound monitoring. JMIR Mhealth Uhealth.

[30] Wang G, Munoz-Ferreras J, Gu C, Li C, Gomez-Garcia R (2014). Linear-frequency-modulated continuous-wave radar for vital sign monitoring.

[31] Huffaker MF, Carchia M, Harris BU, Kethman WC, Murphy TE, Sakarovitch CCD, Qin F, Cornfield DN (2018). Passive nocturnal physiologic monitoring enables early detection of exacerbations in children with asthma. A proof-of-concept study. Am J Respir Crit Care Med.

[32] Seemungal TA, Donaldson GC, Bhowmik A, Jeffries DJ, Wedzicha JA (2000). Time course and recovery of exacerbations in patients with chronic obstructive pulmonary disease. Am J Respir Crit Care Med.

[33] Nandakumar R, Gollakota S, Watson N (2015). Contactless sleep apnea detection on smartphones. Proceedings of the 13th Annual International Conference on Mobile Systems, Applications, and Services.

[34] Nandakumar R, Gollakota S, Sunshine JE (2019). Opioid overdose detection using smartphones. Sci Transl Med.

[35] Wang A, Nguyen D, Sridhar AR, Gollakota S (2021). Using smart speakers to contactlessly monitor heart rhythms. Commun Biol.

[36] Ross C (2019). Amazon Alexa now HIPAA-compliant, allows secure access to data. Stat.

